# Association of changes in lipid levels with changes in vitamin D levels in a real-world setting

**DOI:** 10.1038/s41598-021-01064-1

**Published:** 2021-11-02

**Authors:** Yonghong Li, Carmen H. Tong, Charles M. Rowland, Jeff Radcliff, Lance A. Bare, Michael J. McPhaul, James J. Devlin

**Affiliations:** 1 Quest Diagnostics, San Juan Capistrano, CA USA; 2 UT Southwestern Medical Center, Dallas, TX USA

**Keywords:** Medical research, Epidemiology

## Abstract

In clinical trials, vitamin D supplementation has been reported to reduce serum levels of total cholesterol (TC), low-density lipoprotein cholesterol (LDL-C), and triglycerides (TG) but not high-density lipoprotein cholesterol (HDL-C). In this cohort study we evaluated the association between changes in vitamin D (25-hydroxyvitamin D) and changes in lipid levels in a real-world setting. Changes in lipid levels over a 1-year period were evaluated among individuals whose vitamin D levels increased (group 1) or decreased (group 2) by ≥ 10 ng/mL in year 2018 versus 2017 (cohort 1; n = 5580), in 2019 versus 2018 (cohort 2, n = 6057), or in 2020 versus 2019 (cohort 3, n = 7249). In each cohort, levels of TC, LDL-C, and TG decreased in group 1 and increased in group 2. Between-group differences in average changes in the 3 cohorts ranged from 10.71 to 12.02 mg/dL for TC, from 7.42 to 8.95 mg/dL for LDL-C, and from 21.59 to 28.09 mg/dL for TG. These differences were significant after adjusting for age, sex, race, education, body mass index, blood pressure, smoking status, geographical location, and baseline levels of vitamin D and lipids (*P* < 0.001). Changes in vitamin D levels were not significantly associated with changes in HDL-C levels.

## Introduction

Vitamin D deficiency is common in the general population and has been associated with various health conditions^1,2^. Interest in the health benefits of vitamin D supplementation remains substantial, although evidence for its effect on health outcomes is inconsistent^3–6^. Cardiovascular disease is a case in point: although vitamin D deficiency has been associated with dyslipidemia and cardiovascular disease in observational studies^7–9^, the role of vitamin D supplementation in the management of cardiovascular disease remains debatable^10–12^; supplementation with vitamin D has not been endorsed for primary or secondary prevention of cardiovascular disease.

Individual randomized trials have provided inconclusive evidence for a beneficial effect of vitamin D supplementation on cardiovascular outcomes^11,13^. For example, a recent large randomized trial did not find a lower incidence of cardiovascular events in the group randomized to vitamin D supplementation than in the placebo group, although the authors noted that detection of a benefit (if any) may require longer follow-up or a different patient profile^14^. As an alternative to studies with long follow-up periods—and attendant resource requirements—numerous studies have investigated the effect of vitamin D supplementation on short-term modification of cardiovascular risk factors.

The effect of vitamin D supplementation on lipid profiles has been repeatedly examined in randomized trials and evaluated in meta-analyses^15–19^. A recent meta-analysis reported that vitamin D supplementation improves serum lipid profiles^15^. Combining 41 randomized trials with a total of 3434 participants, this analysis found that vitamin D supplementation reduced the levels of serum total cholesterol (TC), low-density lipoprotein cholesterol (LDL-C), and triglycerides (TG) but not high-density lipoprotein cholesterol (HDL-C). For example, the pooled estimate of the effect from vitamin D supplementation (at a mean dose of 2795 IU per day for a mean duration of 6.9 months) on LDL-C was − 2.92 mg/dL (95% CI − 0.58 to − 5.27) and that on TG was − 6.92 mg/dL (95% CI − 11.97 to − 1.86).

Although this meta-analysis represented 41 clinical trials, it included data from only 1699 individuals receiving vitamin D supplementation. Given the relatively sparse data on the association of vitamin D supplementation with changes in lipid levels, we sought to investigate this relationship in a real-world setting in which supplementation was inferred from year-to-year changes in vitamin D levels. For this study, we retrospectively analyzed vitamin D and lipid levels among working-age adults who had serial monitoring as part of an annual employee health program; unfortunately, this program had no access to information on whether the participants were using statins, a potent modifier of LDL-C levels. In this observational, cross-sectional study, we asked whether year-to-year increases or decreases in serum vitamin D levels were consistently accompanied by changes in serum lipid levels in 3 large study cohorts who had been tested from year 2017 to 2020.

## Results

### Characteristics of study cohorts

Three study cohorts were constructed from the individuals who underwent annual laboratory and biometric testing and whose vitamin D level changed by at least 10 ng/mL (Table 1). The study population totaled 13,989 unique individuals (18,886 person-years) across the 3 study cohorts: 5580 in cohort 1, 6057 in cohort 2, and 7249 in cohort 3. The median (interquartile range) age was 48 (38–56) years in cohort 1, 48 (38–56) in cohort 2, and 49 (39–57) in cohort 3. Cohort 1 had 3906 (70.0%) women; cohort 2 had 4220 (69.7%) women; and cohort 3 had 5034 women (69.4%).

**Table 1:** Baseline characteristics of study cohorts.

Characteristic	Study individuals, no. (%)		
	Cohort 1 (n = 5580)	Cohort 2 (n = 6057)	Cohort 3 (n = 7249)
Testing year compared	2018 vs. 2017	2019 vs. 2018	2020 vs. 2019
Group 1: individuals whose vitamin D increased by ≥ 10 ng/mL^a^	3246 (58.2)	2820 (46.6)	4393 (60.6)
Group 2: individuals whose vitamin D decreased by ≥ 10 ng/mL^a^	2334 (41.8)	3237 (53.4)	2856 (39.4)
**Age, years**			
Median (IQR)	48 (38–56)	48 (38–56)	49 (39–57)
**Sex**			
Male	1674 (30.0)	1837 (30.3)	2215 (30.6)
Female	3906 (70.0)	4220 (69.7)	5034 (69.4)
**Race/ethnicity**			
Asian	684 (15.6)	726 (15.4)	1209 (17.0)
Black	995 (22.7)	1094 (23.2)	1323 (18.6)
Hispanic	539 (12.3)	643 (13.6)	801 (11.2)
Other^b^	30 (0.7)	29 (0.6)	585 (8.2)
White	2128 (48.6)	2232 (47.2)	3212 (45.0)
**Education**			
No college degree	2618 (47.8)	2818 (47.4)	3234 (46.4)
College degree	2862 (52.2)	3128 (52.6)	3730 (53.6)
**Body mass index (kg/m**^**2**^**)**			
≥ 30	1925 (34.5)	2154 (35.6)	2525 (34.9)
< 30	3653 (65.5)	3896 (64.4)	4717 (65.1)
**Blood pressure**			
High	2417 (43.3)	2606 (43.1)	3098 (42.8)
Elevated	817 (14.6)	938 (15.5)	1156 (16.0)
Normal	2343 (42.0)	2505 (41.4)	2987 (41.3)
**Smoking**			
Yes	554 (9.9)	709 (11.7)	813 (11.2)
No	5025 (90.1)	5348 (88.3)	6427 (88.8)
**Geographical location**			
Northeast	1056 (18.9)	1151 (19.1)	1409 (19.5)
Midwest	1020 (18.3)	1083 (18.0)	1256 (17.4)
West	1132 (20.3)	1201 (19.9)	1504 (20.8)
South	2372 (42.5)	2593 (43.0)	3051 (42.3)
**Vitamin D, ng/mL**			
Median (IQR)	32 (23–44)	34 (24–46)	32 (23–42)
**Lipid levels, mg/dL, median (IQR)**			
TC	184 (160–209)	183 (161–208)	184 (161–209)
LDL-C	107 (86–129)	106 (85–127)	108 (86–129)
HDL-C	54 (44–66)	54 (45–66)	54 (46–65)
TG	97 (69–136)	94 (68–136)	95 (69–134)

IQR: interquartile range; TC: total cholesterol; LDL-C: low-density lipoprotein cholesterol; HDL-C: high-density lipoprotein cholesterol; TG: triglycerides.

^a^Comparisons of baseline characteristics between these two groups are reported in Tables S1, S2 and S3.

^b^American Indian or Alaska Native, Native Hawaiian or other Pacific Islander, or 2 or more ethnicities.

Baseline characteristics of groups 1 and 2 in the 3 cohorts are presented in Tables S1, S2 and S3. In each cohort, median baseline vitamin D levels were relatively low in group 1 (26 ng/mL in cohort 1 and 25 ng/mL in cohorts 2 and 3) and high in group 2 (42 ng/mL in cohorts 1 and 2 and 40 ng/mL in cohort 3).

### Association results of changes in vitamin D levels with lipid levels

Among group 1 participants, the mean decrease in TC levels was 5.80 mg/dL in cohort 1, 5.70 mg/dL in cohort 2, and 2.74 mg/dL in cohort 3 (Table 2; Table S4). Conversely, group 2 participants had a mean increase in TC levels of 6.22 mg/dL in cohort 1, 5.98 mg/dL in cohort 2, and 7.96 mg/dL in cohort 3. The difference in average TC changes between these groups ranged from 10.71 to 12.02 mg/dL in the 3 cohorts (*P* < 0.001) (Table 2). These differences remained significant after adjusting for age, sex, race, education, body mass index, blood pressure, smoking status, geographical location, vitamin D level at baseline, and lipid level at baseline (Table 3).

**Table 2:** Changes in lipid levels according to changes in vitamin D levels.

Lipid	Study cohort^a^	Change in lipid levels mean ± SD, mg/dL		Unadjusted difference in the change in lipid levels (95% CI), mg/dL^b^	Unadjusted *P* value
		Vitamin D increased by ≥ 10 ng/mL (group 1)	Vitamin D decreased by ≥ 10 ng/mL (group 2)		
**TC**					
	1	− 5.80 ± 30.52	6.22 ± 27.86	12.02 (10.46–13.59)	< 0.001
	2	− 5.70 ± 30.54	5.98 ± 28.79	11.58 (10.09–13.08)	< 0.001
	3	− 2.74 ± 28.49	7.96 ± 28.20	10.71 (9.37–12.05)	< 0.001
**LDL-C**					
	1	− 5.36 ± 25.85	3.22 ± 23.64	8.59 (7.26–9.91)	< 0.001
	2	− 3.75 ± 26.21	4.69 ± 23.96	8.43 (7.17–9.70)	< 0.001
	3	− 2.24 ± 24.36	5.18 ± 23.80	7.42 (6.28–8.55)	< 0.001
**HDL-C**					
	1	1.14 ± 9.66	0.74 ± 9.52	− 0.40 (− 0.91–1.11)	0.13
	2	0.02 ± 9.06	− 0.46 ± 9.57	− 0.48 (− 0.01 to − 0.95)	0.05
	3	0.10 ± 7.95	0.41 ± 8.77	0.31 (− 0.08 to 0.70)	0.12
**TG**					
	1	− 11.59 ± 51.47	16.50 ± 62.59	28.09 (25.09–31.09)	< 0.001
	2	− 14.07 ± 57.71	12.55 ± 48.99	26.62 (23.93–29.30)	< 0.001
	3	− 4.56 ± 51.23	17.03 ± 61.79	21.59 (18.97–24.21)	< 0.001

TC: total cholesterol; LDL-C: low-density lipoprotein cholesterol; HDL-C: high-density lipoprotein cholesterol; TG: triglycerides; CI: confidence interval; SD: standard deviation.

^a^Cohort 1: 2018 versus 2017; cohort 2: 2019 versus 2018; cohort 3: 2020 versus 2019.

^**b**^Difference between group 2 and group 1.

**Table 3:** Adjusted changes in the lipid levels according to change in vitamin D levels.

Lipid	Study cohort^a^	Model 1^b^		Model 2^c^	
		Adjusted difference in the change in lipid levels (95% CI), mg/dL^d^	Adjusted *P* value	Adjusted difference in the change in lipid levels (95%CI), mg/dL^d^	Adjusted *P* value
**TC**					
	1	11.83 (9.72–13.93)	< 0.001	11.30 (9.37–13.23)	< 0.001
	2	11.74 (9.76–13.72)	< 0.001	11.10 (9.25–12.95)	< 0.001
	3	10.30 (8.72–11.88)	< 0.001	9.53 (8.04–11.02)	< 0.001
**LDL-C**					
	1	8.42 (6.63–10.20)	< 0.001	8.20 (6.55–9.85)	< 0.001
	2	8.21 (6.54–9.89)	< 0.001	8.03 (6.48–9.58)	< 0.001
	3	7.03 (5.69–8.37)	< 0.001	6.22 (4.95–7.49)	< 0.001
**HDL-C**					
	1	− 0.54 (− 1.23 to 0.15)	0.12	− 0.53 (− 1.19 to 0.14)	0.12
	2	− 0.33 (− 0.73 to 0.55)	0.79	− 0.29 (− 0.85 to 0.27)	0.31
	3	0.22 (− 0.25 to 0.69)	0.36	0.48 (0.03–0.93)	0.04
**TG**					
	1	27.88 (24.28–31.48)	< 0.001	24.83 (21.49–28.17)	< 0.001
	2	26.55 (23.09–30.03)	< 0.001	23.61 (20.63–26.60)	< 0.001
	3	21.87 (18.75–25.00)	< 0.001	18.95 (15.95–21.95)	< 0.001

TC: total cholesterol; LDL-C: low-density lipoprotein cholesterol; HDL-C: high-density lipoprotein cholesterol; TG: triglycerides; CI: confidence interval; SD: standard deviation.

^a^Cohort 1: 2018 versus 2017; cohort 2: 2019 versus 2018; cohort 3: 2020 versus 2019.

^b^Adjusted for age, sex, race, education, body mass index, blood pressure, smoking, geographical location, and vitamin D levels.

^c^Adjusted for age, sex, race, education, body mass index, blood pressure, smoking, geographical location, vitamin D and baseline lipid levels.

^d^Difference between the individuals whose vitamin D levels decreased by ≥ 10 ng/mL and the individuals whose vitamin D levels increased by ≥ 10 ng/mL.

Similarly, LDL-C levels among group 1 participants decreased by a mean of 5.36 mg/dL in cohort 1, 3.75 mg/dL in cohort 2, and 2.24 mg/dL in cohort 3 (Table 2). Conversely, LDL-C levels among group 2 participants increased by a mean of 3.22 mg/dL in cohort 1, 4.69 mg/dL in cohort 2, and 5.18 mg/dL in cohort 3. The difference in average LDL-C changes between these groups ranged from 7.42 to 8.59 mg/dL in the 3 cohorts. These differences were significant in both unadjusted analysis and analyses adjusted for baseline characteristics (Tables 2, 3).

Mean TG levels also decreased among group 1 participants: by 11.59 mg/dL in cohort 1, 14.07 mg/dL in cohort 2, and 4.56 mg/dL in cohort 3 (Table 2). Conversely, mean TG levels increased among group 2 participants: by 16.50 mg/dL in cohort 1, 12.55 mg/dL in cohort 2, and 17.03 mg/dL in cohort 3. The difference in average TG changes between these groups ranged from 21.59 to 28.09 mg/dL in the 3 cohorts. These differences were significant in both unadjusted analysis and analyses that adjusted for baseline characteristics (Tables 2, 3).

In all 3 study cohorts, mean HDL-C levels changed only slightly in group 1 and group 2 (Table 2). In unadjusted analysis, the difference in average HDL-C changes between these two groups was statistically significant in cohort 2 (*P* < 0.05) but not in cohort 1 or cohort 3. In analyses adjusting for baseline characteristics, these differences were not significant in any cohort (Table 3).

Among the group 1 and group 2 individuals with baseline vitamin D levels less than 30 ng/mL, changes in lipid levels were also associated with changes in vitamin D (Table S5). In addition, among individuals with year-to-year changes in vitamin D of less than 10 ng/mL, changes in vitamin D levels correlated negatively with changes in TC, LDL-C, and TG (Figure S1). These individuals had negligible changes in HDL-C levels (Figure S1).

## Discussion

In this study we found an association between changes in lipid levels and changes in vitamin D levels in 3 large cohorts of individuals who underwent biometric testing in consecutive years. In these cohorts, individuals who had year-over-year increases in vitamin D levels tended to have corresponding decreases in levels of TC, LDL-C, and TG. Conversely, individuals who had year-over-year decreases in vitamin D levels tended to have increases in TC, LDL-C, and TG levels. Changes in vitamin D levels were not associated with changes in HDL-C levels. These findings are consistent with those of meta-analyses of randomized clinical trials of vitamin D supplementation^15,16,18^.

The magnitudes of the changes in lipid levels associated with changes in vitamin D levels in our study were similar to those reported in the recent meta-analysis^15^. For example, the reduction in LDL-C associated with increased vitamin D levels in our 3 cohorts ranged from 2.74 to 5.80 mg/dL, consistent with the reduction of ~ 3 mg/dL with vitamin D supplementation in the meta-analysis. Similarly, the 4.56- to 14.07-mg/dL reduction in TG associated with increased vitamin D levels in our cohorts is consistent with the ~ 7 mg/dL decrease with vitamin D supplementation reported in the meta-analysis.

Given the effect sizes of the vitamin D supplementation on lipid levels, vitamin D supplementation may be particularly beneficial for those with dyslipidemia. For example, for individuals whose vitamin D levels increased by ≥ 10 ng/mL, compared with those whose vitamin D levels decreased by ≥ 10 ng/mL, the reduction in lipid levels was 7–8 mg/dL for LDL-C, 10–12 mg/dL for TC, and 21–28 mg/dL for TG. However, we note that the changes in LDL-C associated with changes in vitamin D are substantially smaller than those expected from statin use. Nevertheless, the magnitude of the observed changes in LDL-C levels associated with vitamin D levels may be of clinical concern—a concern that may be timely, since vitamin D levels are expected to decline in scenarios such as acutely sun-deprived living conditions^20^. For example, in the current COVID-19 environment, increased time spent indoors could lead to decreased vitamin D levels and increased lipid levels. Consequently, vitamin D supplementation might be warranted to prevent this indirect, negative effect of COVID-19 on cardiovascular health.

This study has several strengths. First, the cohorts included a large number of individuals (18,886 person-years from 13,989 unique individuals). By comparison, the 41 randomized clinical trials included in the meta-analysis comprised a total of only 3434 participants. A recent simulation study highlights the importance of power of the study in vitamin D randomised control trials^21^. Second, we investigated the associations with both increased vitamin D levels and decreased vitamin D levels, an investigation that is not feasible for prospective clinical studies or not done in observations studies before. Ethical considerations would preclude a clinical study intended to reduce vitamin D levels. Third, the sampling period is constrained in each cohort (principally in September to November), minimizing any contribution of seasonal variation in vitamin D levels, a factor identified as a potential confounder in clinical trials conducted across a broader period of time. Finally, in clinical trials, a change in vitamin D levels by a certain degree may not be achievable for everyone because of variations in intrinsic response to^22^ and compliance to vitamin D supplementation. In this regard, novel vitamin D clinical trials involving vitamin D supplementation but based primarily on vitamin D concentrations have been proposed to more accurately assess how vitamin D concentrations affect health outcomes^23^.

Unlike randomized clinical trials, this observational study made no intervention but instead evaluated the association between changes in lipid levels and changes in vitamin D levels in a population assessed annually; we do not know the reason for the changes in vitamin D and lipid levels and are not able to establish causality. However, 25-hydroxyvitamin D is known to inhibit sterol regulatory element-binding protein (SREBP), a master regulator of lipogenesis^24^. This could be a possible mechanism by which increased vitamin D levels modulate levels of TC, LDL-C, and TG. Given the similarity of our findings to results from randomized clinical trials^15^ and the potential role of vitamin D in cholesterol synthesis and lipid profiles^25,26^, our findings provide additional support for the concept that vitamin D levels modulate levels of TC, LDL-C, and TG.

This study has several limitations. One limitation is that unobserved covariates could have contributed to the association between changes in lipid levels and changes in vitamin D levels. For example, we did not have information on statin use among the study participants and therefore could not include statin use in the association models. Another limitation is that this population of employer health program participants may not be representative of the general US population.

In summary, in this study of 3 large cohorts of working-age adults, we found consistent associations between changes in vitamin D levels and changes in lipid levels. These findings are consistent with those of a meta-analysis of randomized clinical trials demonstrating that vitamin D supplementation improves lipid profiles. Furthermore, our findings support that reductions in vitamin D levels are associated with worsening lipid profiles.

## Methods

### Study setting and design

All methods were carried out in accordance with relevant guidelines and regulations: the data used for this analysis were de-identified as required by the HIPAA Privacy Rule (45 CFR 164.514). The Western Institutional Review Board determined that this study was exempt from IRB review and the need for informed consent because it was a retrospective analysis of de-identified data. Dr. Li had full access to all the data in the study and takes responsibility for the integrity of the data and the accuracy of the data analysis.

This study analyzed changes in lipid levels and changes in vitamin D levels among individuals who participated in an employer-sponsored biometric testing program that included collection of blood samples by venous blood draw for laboratory testing. This program is offered annually to employees and spouses of Quest Diagnostics, a clinical laboratory with workforce members in every state of the United States. Participants receive financial incentives to participate and refrain from tobacco use. Collections typically occur in September to November of each year.

**Figure 1:**
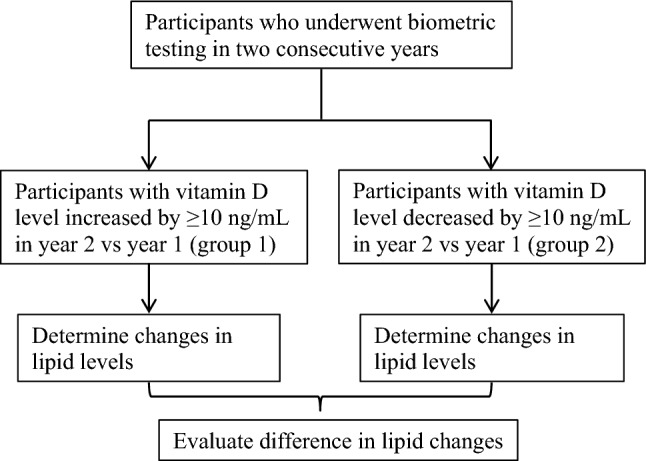
Study design. Selection process of individuals into the study cohort and analysis plan are presented.

Our study cohorts consisted of individuals whose vitamin D levels increased (group 1) or decreased (group 2) by at least 10 ng/mL year-over-year (Fig. 1). The decision to use 10 ng/mL as the threshold for increased vitamin D levels was based on findings from the VITamin D and OmegA-3 TriaL^14^, in which the mean 1-year 25-hydroxyvitamin D increased by 11.9 ng/mL in a subset of the study participants in the active vitamin D supplementation arm^27^. Three study cohorts were selected from participants who had serial testing in 2017 and 2018 (cohort 1), 2018 and 2019 (cohort 2), or 2019 and 2020 (cohort 3). All participants meeting criteria for group 1 or 2 were included.

## Measurements

All tests and measurements were performed by Quest Diagnostics. Fasting for 8–12 h before blood sample collection was advised to the individuals undergoing biometric testing. Total 25-hydroxyvitamin D, the major circulating form of vitamin D, was measured using a chemiluminescent immunoassay (DiaSorin LIAISON1XL 25-hydroxyvitamin D, total) or a laboratory-developed test based on liquid chromatograph/tandem mass spectrometry. TC, TG and HDL-C were directly measured and LDL-C was calculated. TC and TG levels were determined by spectrophotometry using reagents from Beckman Coulter, and the HDL-C levels were determined using reagent from Roche Diagnostics. Blood pressure was measured with an automatic upper-arm monitor before blood sample collection. Normal blood pressure was defined as having a systolic blood pressure of < 120 mmHg and a diastolic blood pressure of < 80 mmHg. Elevated blood pressure was defined as having a systolic blood pressure of 120 to 129 mmHg and a diastolic blood pressure of < 80 mmHg. High blood pressure was defined as having a systolic blood pressure of ≥ 130 mmHg or a diastolic blood pressure of ≥ 80 mmHg. Smoking status was determined by a serum cotinine assay; individuals who had serum cotinine level ≥ 10 ng/mL were classified as current smokers. Obesity was defined as having a body mass index (BMI) of ≥ 30 kg/m^2^. The geographical locations were defined by the Census Bureau-designated regions: West, South, Midwest, and Northeast.

Ethnicity was self-reported and categorized as Asian, Black, Hispanic, White, or other (those who self-reported as American Indian or Alaska Native, Native Hawaiian or other Pacific Islander, or two or more ethnicities). Educational attainment was also self-reported and categorized as having a college degree or not.

### Statistical analysis

Differences in the biochemical and demographic characteristics according to changes in vitamin D levels (increased by ≥ 10 ng/mL vs. decreased by ≥ 10 ng/mL) were assessed with the Wilcoxon rank-sum test for continuous variables and by the *χ*^2^ test for discrete variables. Linear regression analyses were carried out to estimate the association of vitamin D change status (independent variable) with change in serum lipids (dependent variables: TC, LDL-C, TG, and HDL-C), with or without controlling for age, sex, race, education, body mass index, blood pressure, smoking status, geographical location, and baseline vitamin D level. Further adjustment for the lipid level was also performed. Sex, race, education, BMI, blood pressure, smoking status, and geographical location were coded as categorical variables in the linear regression model; the cutoffs are described in the measurement section and the supplemental Tables. Age and baseline vitamin D and lipid levels were coded as a continuous variable in the linear regression model. All analyses were performed using SAS version 9.4 (https://support.sas.com/software/94/; SAS Institute).

## Supplementary Information

Supplementary Information.
