# Supplementary material for: Association of changes in lipid levels with changes in vitamin D levels in a real-world setting
Source: Sci Rep. 2021 Nov 2;11:21536. doi: 10.1038/s41598-021-01064-1 (PMC8563916; doi:10.1038/s41598-021-01064-1)

**Supplementary information**

**Association of changes in lipid levels with changes in vitamin D levels in a real-world setting**

Yonghong Li^1*^, Carmen H. Tong^1^, Charles M. Rowland^1^, Jeff Radcliff^1^, Lance A. Bare^1^, Michael J. McPhaul^1,2^, James J. Devlin^1^

^1^Quest Diagnostics, San Juan Capistrano, California, USA

^2^UT Southwestern Medical Center, Dallas, Texas, USA

**Table S1** Baseline characteristics according to changes in the vitamin D levels in study cohort 1 (2018 vs 2017)

**Table S2** Baseline characteristics according to changes in the vitamin D levels in study cohort 2 (2019 vs 2018)

**Table S3** Baseline characteristics according to changes in the vitamin D levels in study cohort 3 (2020 vs 2019)

**Table S4** Changes in lipid levels relative to the standard deviation according to changes in vitamin D levels

**Table S5** Changes in lipid levels according to changes in vitamin D levels among individuals with baseline vitamin D levels less than 30 ng/mL

**Figure S1** Average changes in lipid levels according to changes in vitamin D among participants with year-to-year vitamin D changes of less than 10 ng/mL

**Table S1** Baseline characteristics according to changes in the vitamin D levels in study cohort 1 (2018 vs 2017)

|  | **Study individuals, No. (%)** | |  |  |
| --- | --- | --- | --- | --- |
| **Characteristic** | **Vitamin D increased by ≥10 ng/mL in 2018 vs. 2017 (group 1, n=3246)** | **Vitamin D decreased by ≥ 10 ng/mL in 2018 vs. 2017 (group 2, n=2334)** | **P value**^†^ | **Standardized difference** |
| Age (y) |  |  |  |  |
| Median (IQR) | 49 (40-57) | 48 (38-56) | .21 | 0.04 |
| Sex |  |  |  |  |
| Male | 1008 (31.1) | 666 (28.5) | .04 | 0.06 |
| Female | 2238 (68.9) | 1668 (71.5) |  |  |
| Race/Ethnicity |  |  |  |  |
| Asian | 421 (16.7) | 263 (14.2) | .24 | 0.07 |
| Black | 575 (22.8) | 420 (22.7) |  |  |
| Hispanic | 302 (12.0) | 237 (12.8) |  |  |
| Other^*^ | 17 (0.7) | 13 (0.7) |  |  |
| White | 1208 (47.9) | 920 (49.6) |  |  |
| Education |  |  |  |  |
| No college degree | 1486 (46.6) | 1132 (49.4) | .04 | 0.06 |
| College degree | 1704 (53.4) | 1158 (50.6) |  |  |
| Body mass index, kg/m^2^ |  |  |  |  |
| ≥30 | 1168 (36.0) | 757 (32.5) | .006 | 0.09 |
| <30 | 2078 (64.0) | 1575 (67.5) |  |  |
| Blood pressure |  |  |  |  |
| High | 1440 (44.4) | 977 (41.9) | .08 | 0.06 |
| Elevated | 483 (14.9) | 334 (14.3) |  |  |
| Normal | 1323 (40.8) | 1020 (43.8) |  |  |
| Smoking |  |  |  |  |
| Yes | 299 (9.2) | 255 (10.9) | .04 | 0.06 |
| No | 2946 (90.8) | 2079 (89.1) |  |  |
| Geographical location |  |  |  |  |
| Northeast | 651 (20.1) | 405 (17.4) | .008 | 0.09 |
| Midwest | 560 (17.3) | 460 (19.7) |  |  |
| West | 677 (20.9) | 455 (19.5) |  |  |
| South | 1358 (41.8) | 1014 (43.4) |  |  |
| Vitamin D, ng/mL |  |  |  |  |
| Median (IQR) | 26 (18-34) | 42 (34-52) | <.001 | 1.16 |
| Lipid levels, mg/dL, Median (IQR) |  |  |  |  |
| TC | 186 (161-212) | 182 (158-206) | <.001 | 0.13 |
| LDL-C | 108 (87-130) | 104 (83-127) | <.001 | 0.14 |
| HDL-C | 53 (44-65) | 55 (45-67) | <.001 | 0.09 |
| TG | 102 (71-146) | 92 (67-125) | <.001 | 0.26 |

IQR: interquartile range; TC: total cholesterol; LDL-C: low-density lipoprotein cholesterol; HDL-C: high-density lipoprotein cholesterol; TG: triglycerides.

^*^American Indian or Alaska Native, Native Hawaiian or other Pacific Islander, or 2 or more ethnicities

^†^Unadjusted results from Wilcoxon rank-sum test for continuous variables and from the χ^2^ test for discrete variables

**Table S2** Baseline characteristics according to changes in the vitamin D levels in study cohort 2 (2019 vs 2018)

|  | **Study individuals, No. (%)** | |  |  |
| --- | --- | --- | --- | --- |
| **Characteristic** | **Vitamin D increased by ≥10 ng/mL in 2019 vs. 2018 (group 1, n=2820)** | **Vitamin D decreased by ≥ 10 ng/mL in 2019 vs. 2018 (group 2, n=3237)** | **P value**^†^ | **Standardized difference** |
| Age (y) |  |  |  |  |
| Median (IQR) | 49 (40-57) | 48 (38-57) | .30 | 0.03 |
| Sex |  |  |  |  |
| Male | 871 (30.9) | 966 (29.8) | .38 | 0.02 |
| Female | 1949 (69.1) | 2271 (70.2) |  |  |
| Race/Ethnicity |  |  |  |  |
| Asian | 346 (15.5) | 380 (15.2) | .15 | 0.08 |
| Black | 528 (23.7) | 566 (22.7) |  |  |
| Hispanic | 322 (14.4) | 321 (12.9) |  |  |
| Other^*^ | 17 (0.8) | 12 (0.5) |  |  |
| White | 1016 (45.6) | 1216 (48.7) |  |  |
| Education |  |  |  |  |
| No college degree | 1306 (47.3) | 1512 (47.5) | .89 | 0.003 |
| College degree | 1455 (52.7) | 1673 (52.5) |  |  |
| Body mass index, kg/m^2^ |  |  |  |  |
| ≥30 | 1097 (38.9) | 1057 (32.7) | <.001 | 0.16 |
| <30 | 1720 (61.1) | 2176 (67.3) |  |  |
| Blood pressure |  |  |  |  |
| High | 1294 (46) | 1312 (40.6) | <.001 | 0.11 |
| Elevated | 420 (14.9) | 518 (16.0) |  |  |
| Normal | 1102 (39.1) | 1403 (43.4) |  |  |
| Smoking |  |  |  |  |
| Yes | 337 (12.0) | 372 (11.5) | .58 | 0.01 |
| No | 2483 (88.0) | 2865 (88.5) |  |  |
| Geographical location |  |  |  |  |
| Northeast | 517 (18.4) | 634 (19.7) | <.001 | 0.12 |
| Midwest | 458 (16.3) | 625 (19.4) |  |  |
| West | 546 (19.4) | 655 (20.3) |  |  |
| South | 1288 (45.9) | 1305 (40.5) |  |  |
| Vitamin D, ng/mL |  |  |  |  |
| Median (IQR) | 25 (18-33) | 42 (33-52) | <.001 | 1.21 |
| Lipid levels, mg/dL,  Median (IQR) |  |  |  |  |
| TC | 185 (163-211) | 182 (159-207) | <.001 | 0.11 |
| LDL-C | 108 (87-125) | 104 (84-125) | <.001 | 0.11 |
| HDL-C | 53 (44-65) | 55 (45-67) | <.001 | 0.10 |
| TG | 100 (71-147) | 90 (67-127) | <.001 | 0.25 |

IQR: interquartile range; TC: total cholesterol; LDL-C: low-density lipoprotein cholesterol; HDL-C: high-density lipoprotein cholesterol; TG: triglycerides.

^*^American Indian or Alaska Native, Native Hawaiian or other Pacific Islander, or 2 or more ethnicities

^†^Unadjusted results from Wilcoxon rank-sum test for continuous variables and from the χ^2^ test for discrete variables

**Table S3** Baseline characteristics according to changes in the vitamin D levels in study cohort 3 (2020 vs 2019)

|  | **Study individuals, No. (%)** | |  |  |
| --- | --- | --- | --- | --- |
| **Characteristic** | **Vitamin D increased by ≥10 ng/mL in 2020 vs. 2019 (group 1, n=4393)** | **Vitamin D decreased by ≥ 10 ng/mL in 2020 vs. 2019 (group 2, n=2856)** | **P value**^†^ | **Standardized difference** |
| Age (y) |  |  |  |  |
| Median (IQR) | 49 (40-57) | 47 (37-56) | <.001 | 0.15 |
| Sex |  |  |  |  |
| Male | 1379 (31.4) | 836 (29.3) | .06 | 0.05 |
| Female | 3014 (68.6) | 2020 (70.7) |  |  |
| Race |  |  |  |  |
| Asian | 801 (18.5) | 408 (14.6) | <.001 | 0.18 |
| Black | 878 (20.3) | 445 (15.9) |  |  |
| Hispanic | 467 (10.8) | 334 (11.9) |  |  |
| Other^*^ | 353 (8.2) | 232 (8.3) |  |  |
| White | 1831 (42.3) | 1381 (49.3) |  |  |
| Education |  |  |  |  |
| No college degree | 1937 (45.9) | 1297 (47.3) | .24 | 0.03 |
| College degree | 2285 (54.1) | 1445 (52.7) |  |  |
| Body mass index, kg/m^2^ |  |  |  |  |
| ≥30 | 1688 (38.5) | 837 (29.3) | <.001 | 0.24 |
| <30 | 2700 (61.5) | 2017 (70.7) |  |  |
| Blood pressure |  |  |  |  |
| High | 1984 (45.2) | 1114 (39.0) | <.001 | 0.14 |
| Elevated | 706 (16.1) | 450 (15.8) |  |  |
| Normal | 1698 (38.7) | 1289 (45.2) |  |  |
| Smoking |  |  |  |  |
| Yes | 499 (11.4) | 314 (11.0) | .61 | 0.01 |
| No | 3885 (88.6) | 2542 (89.0) |  |  |
| Geographical location |  |  |  |  |
| Northeast | 1025 (23.4) | 384 (13.5) | <.001 | 0.27 |
| Midwest | 699 (16.0) | 557 (19.6) |  |  |
| West | 842 (19.2) | 662 (23.3) |  |  |
| South | 1814 (41.4) | 1237 (43.6) |  |  |
| Vitamin D, ng/mL |  |  |  |  |
| Median (IQR) | 25 (18-35) | 40 (33-49) | <.001 | 1.04 |
| Lipid levels, mg/dL,  Median (IQR) |  |  |  |  |
| TC | 186 (163-211) | 181 (157-205) | <.001 | 0.15 |
| LDL-C | 108 (88-131) | 103 (82-124) | <.001 | 0.20 |
| HDL-C | 53 (45-64) | 56 (47-67) | <.001 | 0.21 |
| TG | 101 (72-144) | 87 (65-120) | <.001 | 0.30 |

IQR: interquartile range; TC: total cholesterol; LDL-C: low-density lipoprotein cholesterol; HDL-C: high-density lipoprotein cholesterol; TG: triglycerides.

^*^American Indian or Alaska Native, Native Hawaiian or other Pacific Islander, or 2 or more ethnicities

**Table S4** Changes in lipid levels relative to the standard deviation according to changes in vitamin D levels

| **Lipid** | **Study cohort*** | **Vitamin D increased by ≥10 ng/mL (group 1)** | **Vitamin D decreased by ≥10 ng/mL (group 2)** |
| --- | --- | --- | --- |
| TC |  |  |  |
|  | 1 | -0.19 | 0.22 |
|  | 2 | -0.19 | 0.19 |
|  | 3 | -0.10 | 0.33 |
| LDL-C |  |  |  |
|  | 1 | -0.21 | 0.15 |
|  | 2 | -0.15 | 0.19 |
|  | 3 | -0.09 | 0.25 |
| HDL-C |  |  |  |
|  | 1 | 0.12 | 0.08 |
|  | 2 | 0.00 | -0.05 |
|  | 3 | 0.01 | 0.05 |
| TG |  |  |  |
|  | 1 | -0.21 | 0.19 |
|  | 2 | -0.23 | 0.23 |
|  | 3 | -0.08 | 0.23 |

TC: total cholesterol; LDL-C: low-density lipoprotein cholesterol; HDL-C: high-density lipoprotein cholesterol; TG: triglycerides

^*^Cohort 1: 2018 vs. 2017; cohort 2: 2019 vs. 2018; cohort 3: 2020 vs. 2019

**Table S5** Changes in lipid levels according to changes in vitamin D levels among individuals with baseline vitamin D levels less than 30 ng/mL

| **Lipid** | **Study cohort**^*^ | **Change in lipid levels mean±SD, mg/dL** | | **Unadjusted P-value** | **Adjusted P-value**^†^ |
| --- | --- | --- | --- | --- | --- |
|  |  | **Vitamin D increased by ≥10 ng/mL (group 1)** | **Vitamin D decreased by ≥10 ng/mL (group 2)** |  |  |
| TC |  |  |  |  |  |
|  | 1 | -5.36±30.99 | 3.87±27.96 | <.001 | <.001 |
|  | 2 | -5.40±30.37 | 6.02±31.39 | <.001 | <.001 |
|  | 3 | -2.40±28.68 | 7.73±24.41 | <.001 | <.001 |
| LDL-C |  |  |  |  |  |
|  | 1 | -4.88±26.15 | 1.35±22.10 | <.001 | .01 |
|  | 2 | -3.58±25.70 | 4.24±24.70 | <.001 | <.001 |
|  | 3 | -2.02±24.69 | 5.31±20.87 | <.001 | <.001 |
| HDL-C |  |  |  |  |  |
|  | 1 | 1.06±9.52 | -0.35±8.98 | .01 | .51 |
|  | 2 | 0.33±8.84 | -0.41±9.60 | .12 | .67 |
|  | 3 | 0.23±8.00 | -0.17±8.38 | .34 | .49 |
| TG |  |  |  |  |  |
|  | 1 | -11.26 ± 54.43 | 21.96±86.13 | <.001 | <.001 |
|  | 2 | -15.17±62.14 | 15.55±55.32 | <.001 | <.001 |
|  | 3 | -4.53±54.40 | 19.37±73.95 | <.001 | <.001 |

TC: total cholesterol; LDL-C: low-density lipoprotein cholesterol; HDL-C: high-density lipoprotein cholesterol; TG: triglycerides; CI: confidence interval; SD: standard deviation

^*^Cohort 1: 2018 vs. 2017; cohort 2: 2019 vs. 2018; cohort 3: 2020 vs. 2019

^†^Adjusted for age, sex, race, education, body mass index, blood pressure, smoking, geographical location, vitamin D and baseline lipid levels

**Figure S1**

**Legend**: Average changes in lipid levels according to changes in vitamin D among participants with year-to-year vitamin D changes of less than 10 ng/mL. These results were derived from 22,810 individuals who participated in wellness screening in both 2017 and 2018 and had vitamin D changes in the range of -9 ng/mL to 9 ng/mL. TC: total cholesterol; LDL-C: low-density lipoprotein cholesterol; HDL-C: high-density lipoprotein cholesterol; TG: triglycerides.


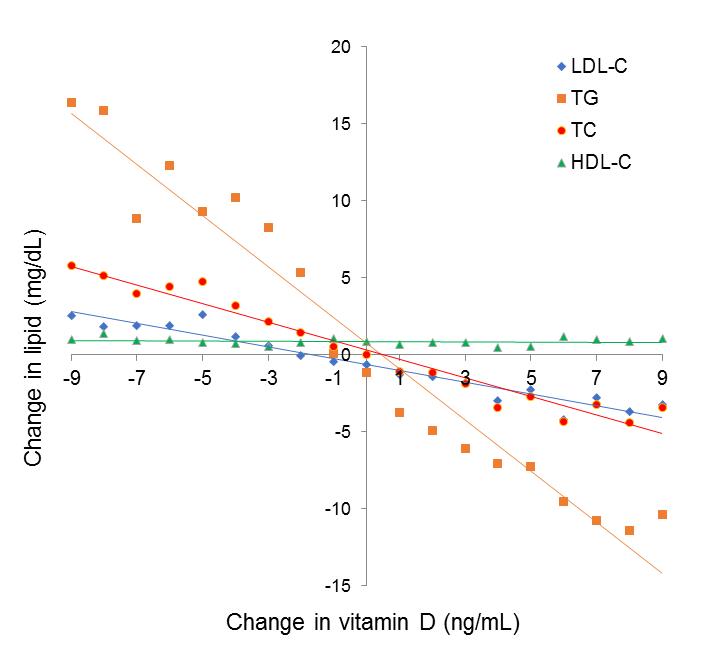

Supplement: Supplementary file 1 — Supplementary Information. [file 41598_2021_1064_MOESM1_ESM.docx]
